# NAD^+^ Metabolism, Metabolic Stress, and Infection

**DOI:** 10.3389/fmolb.2021.686412

**Published:** 2021-05-19

**Authors:** Benjamin Groth, Padmaja Venkatakrishnan, Su-Ju Lin

**Affiliations:** Department of Microbiology and Molecular Genetics, College of Biological Sciences, University of California, Davis, Davis, CA, United States

**Keywords:** *de novo* NAD^+^ synthesis, kynurenine metabolites, quinolinic acid, budding yeast, siutuin, immune response, infection

## Abstract

Nicotinamide adenine dinucleotide (NAD^+^) is an essential metabolite with wide-ranging and significant roles in the cell. Defects in NAD^+^ metabolism have been associated with many human disorders; it is therefore an emerging therapeutic target. Moreover, NAD^+^ metabolism is perturbed during colonization by a variety of pathogens, either due to the molecular mechanisms employed by these infectious agents or by the host immune response they trigger. Three main biosynthetic pathways, including the *de novo* and salvage pathways, contribute to the production of NAD^+^ with a high degree of conservation from bacteria to humans. *De novo* biosynthesis, which begins with l-tryptophan in eukaryotes, is also known as the kynurenine pathway. Intermediates of this pathway have various beneficial and deleterious effects on cellular health in different contexts. For example, dysregulation of this pathway is linked to neurotoxicity and oxidative stress. Activation of the *de novo* pathway is also implicated in various infections and inflammatory signaling. Given the dynamic flexibility and multiple roles of NAD^+^ intermediates, it is important to understand the interconnections and cross-regulations of NAD^+^ precursors and associated signaling pathways to understand how cells regulate NAD^+^ homeostasis in response to various growth conditions. Although regulation of NAD^+^ homeostasis remains incompletely understood, studies in the genetically tractable budding yeast *Saccharomyces cerevisiae* may help provide some molecular basis for how NAD^+^ homeostasis factors contribute to the maintenance and regulation of cellular function and how they are regulated by various nutritional and stress signals. Here we present a brief overview of recent insights and discoveries made with respect to the relationship between NAD^+^ metabolism and selected human disorders and infections, with a particular focus on the *de novo* pathway. We also discuss how studies in budding yeast may help elucidate the regulation of NAD^+^ homeostasis.

## Introduction

NAD^+^ and its reduced form NADH are essential redox cofactors with wide-ranging and significant roles in the cell. NAD^+^ is well known as an electron carrier in core metabolic pathways, as in glycolysis, oxidative phosphorylation, and β-oxidation of fatty acids. NAD^+^ also serves as a co-substrate in several non-redox reactions, such as sirtuin-mediated protein deacetylation, which regulates the activity of many target proteins including histones. Sirtuins are Sir2 family proteins (class III histone deacetylases, HDACs) the activity of which depends on NAD^+^ (Imai et al., 2000; Landry et al., 2000; Smith et al., 2000). In addition, NAD^+^ is also consumed by the poly-ADP-ribose polymerases (PARPs) for the purpose of ADP-ribosylation during base excision repair of single-strand breaks (Kraus 2015), and by CD38, a cell surface NAD^+^ glycohydrolase (an ectonucleotidase) (Chini et al., 2020; Katsyuba et al., 2020; Covarrubias et al., 2021). Although its biological function is not completely understood, CD38 activation has been shown to cause NAD^+^ decline in several cell/tissue types and CD38 inhibition is able to restore NAD^+^ (Camacho-Pereira et al., 2016; Tarragó et al., 2018). Consequently, NAD^+^ has a multifarious and highly important influence on cellular health, affecting an extensive suite of processes, including: DNA repair, central metabolism, circadian rhythms, meiosis and lifespan (Imai and Guarente, 2014; Kato and Lin, 2014; Nikiforov et al., 2015; Chini et al., 2016; Yoshino et al., 2018; Okabe et al., 2019; Castro-Portuguez and Sutphin, 2020). Owing to its centrality in cellular homeostasis, defects in NAD^+^ metabolism are often associated with a variety of disease states, seen in diabetes, neurological disorders, and various cancers (Schwarcz et al., 2012; Imai and Guarente, 2014; Cantó et al., 2015; Garten et al., 2015; Nikiforov et al., 2015; Verdin, 2015; Cheng et al., 2016; Chini et al., 2016; Yang and Sauve, 2016; Williams et al., 2017; Liu et al., 2018; Poyan Mehr et al., 2018; Yaku et al., 2018; Yoshino et al., 2018; Okabe et al., 2019; Chini et al., 2020; Covarrubias et al., 2020; Katsyuba et al., 2020; Covarrubias et al., 2021). Administration of NAD^+^ precursors such as nicotinamide mononucleotide (NMN), nicotinamide (NAM), nicotinic acid riboside (NaR), nicotinamide riboside (NR), and dihydronicotinamide riboside (NRH) has been shown to increase NAD^+^ levels and ameliorate associated deficiencies in various model systems and in humans (Belenky et al., 2007; Brown et al., 2014; Cantó et al., 2015; Edwards et al., 2015; Garten et al., 2015; Verdin, 2015; Chini et al., 2016; Lin et al., 2016; Ryu et al., 2016; Yang and Sauve, 2016; Zhang et al., 2016; Williams et al., 2017; Katsyuba et al., 2018; Liu et al., 2018; Meng et al., 2018; Mitchell et al., 2018; Poyan Mehr et al., 2018; Rajman et al., 2018; Yoshino et al., 2018; Sambeat et al., 2019; Vannini et al., 2019; Yang et al., 2019; Castro-Portuguez and Sutphin, 2020; Hui et al., 2020; Pirinen et al., 2020). However, the molecular mechanisms underlying the beneficial effects of NAD^+^ precursor treatments are not completely understood.

**FIGURE 1:**
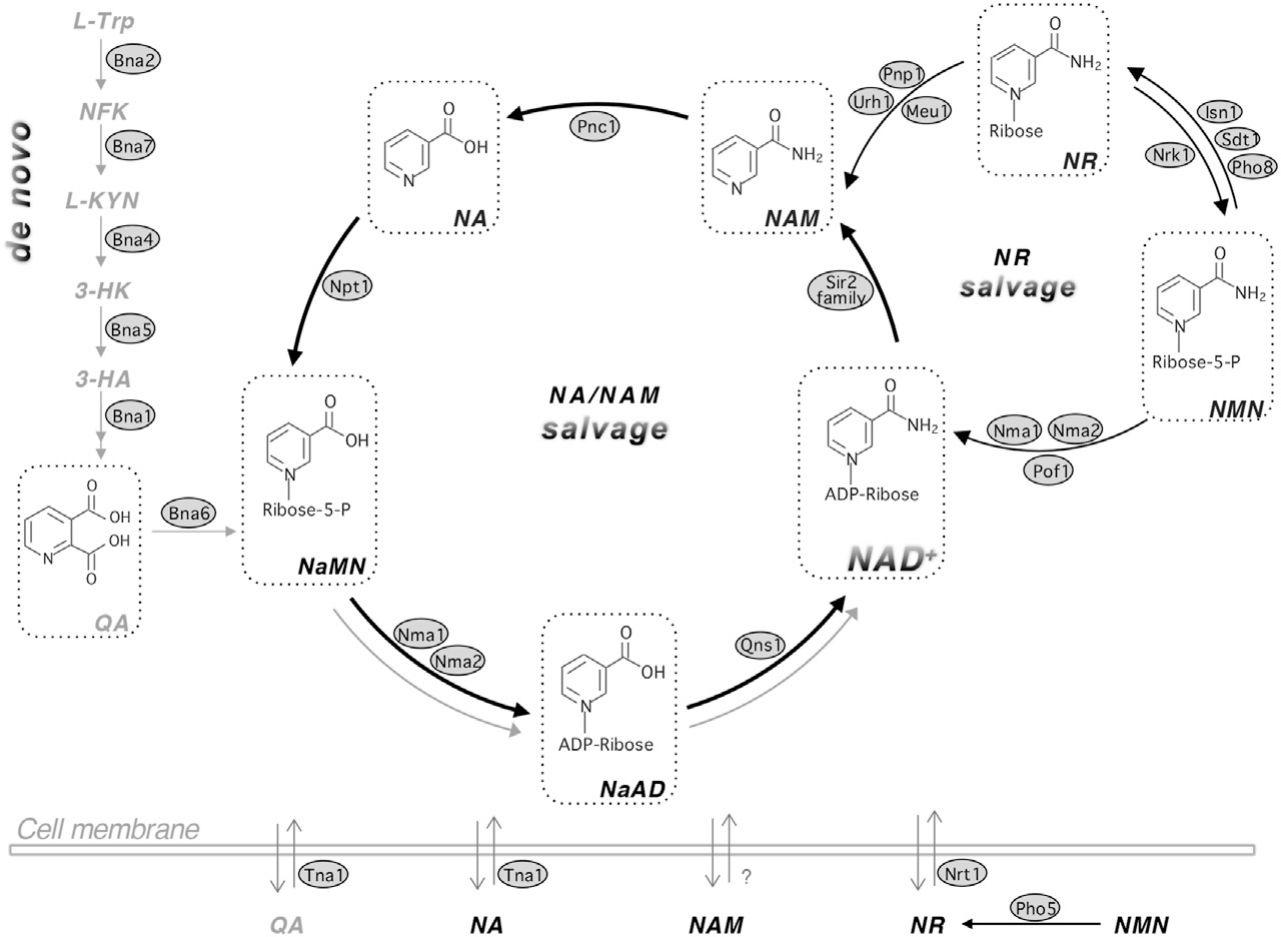
The NAD^+^ biosynthetic pathways in yeast *Saccharomyces cerevisiae*. Budding yeast maintains the NAD^+^ pool by three major pathways: *de novo* synthesis (*left panel*), NA/NAM salvage (*center circle*) and NR salvage (*right circle*). NAD^+^ can be made by salvaging precursors such as NA, NAM and NR or by *de novo* synthesis from *L-TRP*. Yeast cells also release and re-uptake these precursors. The *de novo* NAD^+^ synthesis (*left panel*) is mediated by Bna proteins (Bna2,7,4,5,1) leading to the production of NaMN. This pathway is inhibited by Hst1 when NAD^+^ is abundant and therefore is shown in gray color. The NA/NAM salvage pathway (*center circle*) also produces NaMN, which is then converted to NaAD and NAD^+^ by Nma1/2 and Qns1, respectively. NAD^+^ biosynthesis from the NA/NAM salvage and *de novo* pathways converges at the formation of NaMN. The NA/NAM salvage pathway is highlighted with bold black arrows because most yeast growth media contain abundant NA. In mammals, NAM is converted to NMN by NAM phosphoribosyl transferase, NAMPT or converted to NA by microbial nicotimidase in the gut. NR salvage (*right circle*) connects to the NA/NAM salvage pathway by Urh1, Pnp1 and Meu1. NR turns into NMN by Nrk1, which is then converted to NAD^+^ by Nma1, Nma2 and Pof1. For simplicity, NaR (nicotinic acid riboside) and NRH (reduced NR) salvage pathways, which overlap with NR salvage, are not shown in this figure. Abbreviations of NAD^+^ intermediates are shown in bold and italicized. ***NA***, nicotinic acid. ***NAM***, nicotinamide. ***NR***, nicotinamide riboside. ***QA***, quinolinic acid. ***L-TRP***, l-tryptophan. ***NFK***, N-formylkynurenine. ***L-KYN***, l-kynurenine. ***3-HK***, 3-hydroxykynurenine. ***3-HA***, 3-hydroxyanthranilic acid. ***NaMN***, nicotinic acid mononucleotide. ***NaAD***, deamido-NAD^+^. ***NMN***, nicotinamide mononucleotide. Abbreviations of protein names are shown in shaded ovals. Bna2, tryptophan 2,3-dioxygenase. Bna7, kynurenine formamidase. Bna4, kynurenine 3-monooxygenase. Bna5, kynureninase. Bna1, 3-hydroxyanthranilate 3,4-dioxygenase. Bna6, quinolinic acid phosphoribosyl transferase. Nma1/2, NaMN/NMN adenylyltransferase (NMNAT). Qns1, glutamine-dependent NAD^+^ synthetase. Npt1, nicotinic acid phosphoribosyl transferase. Pnc1, nicotinamide deamidase. Sir2 family, NAD^+^-dependent protein deacetylases. Urh1, Pnp1 and Meu1, nucleosidases. Nrk1, NR kinase. Isn1 and Sdt1, nucelotidases. Pho8 and Pho5, phosphatases. Pof1, NMN adenylyltransferase (NMNAT). Tna1, NA and QA transporter. Nrt1, NR transporter.

**FIGURE 2:**
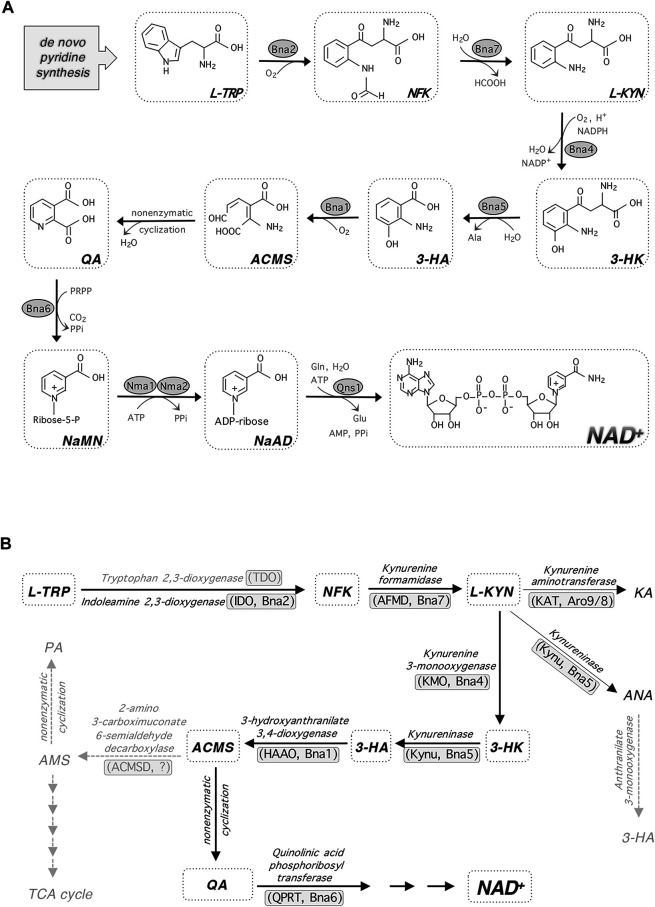
*de novo* NAD^+^ synthesis and kynurenine metabolism. **(A)** *de novo* NAD^+^ synthesis starts with L-TRP in budding yeast and most eukaryotes. Enzymatic reactions of Bna2, Bna4 and Bna1 requires molecular oxygen. **(B)** In addition to feeding into NAD^+^ synthesis, L-KYN is also converted to KA and ANA. The enzymes mediating each step are shown along with their abbreviations in parentheses (*left, in mammals; right, in yeast*). Steps shown in gray indicate that they may be missing in yeast. Abbreviations of metabolites and enzymes: HCOOH, formic acid. Ala, alanine. PRPP, phosphoribosyl pyrophosphate. PPi, pyrophosphate. Gln, glutamine. Glu, glutamic acid. ACMS, 2-amino-3-carboximuconate-6-semialdehyde. KA, kynurenic acid. ANA, anthranilic acid. PA, picolinic acid. AMS, aminomuconic semialdehyde. ACMSD, ACM decarboxylase.

NAD^+^ metabolism is an emerging therapeutic target for a number of human disorders (Braidy and Grant, 2017; Katsyuba et al., 2018; Liu et al., 2018; Pellicciari et al., 2018; Poyan Mehr et al., 2018; Katsyuba et al., 2020; Pirinen et al., 2020; Covarrubias et al., 2021). Due to the redundancy and interconnection of NAD^+^ biosynthesis pathways (Figure 1), supplementations of specific NAD^+^ precursors often need to be combined with the use of genetic interventions and chemical inhibitors of specific NAD^+^ biosynthesis steps to help channel the flow of precursors through a more efficient NAD^+^ synthesis route (Braidy and Grant, 2017; Katsyuba et al., 2018; Liu et al., 2018; Poyan Mehr et al., 2018). In addition to NAD^+^ deficiency, aberrant accumulation of specific NAD^+^ metabolites may alter cellular function. For example, kynurenine (KYN) pathway metabolites, which are produced during *de novo* NAD^+^ biosynthesis (Figure 2), have been linked to brain disorders independent of NAD^+^ levels (Schwarcz et al., 2012; Amaral et al., 2013; Badawy, 2017; Braidy and Grant, 2017; Katsyuba et al., 2018; Castro-Portuguez and Sutphin, 2020). Interestingly, several pathogens also appear to target the NAD^+^ metabolic network upon infection including *Mycobacterium tuberculosis* (Sun et al., 2015; Pajuelo et al., 2020), *Aspergillus fumigatus* (Choera et al., 2017; Zelante et al., 2021), *Toxoplasma gondii* (Majumdar et al., 2019; Abo-Al-Ela, 2020), SARS-CoV-2 (COVID-19) (Heer et al., 2020; Martorana et al., 2020; Thomas et al., 2020), and HIV (Bipath et al., 2015; Kardashian et al., 2019). Aberrations of NAD^+^ and KYN metabolites homeostasis are observed during infections, which may play a role in the modulation of host’s immune response and inflammatory signaling.

NAD^+^ levels also appear to decline with age, which may render the elderly more susceptible to various infections as well as age-associated disorders (Belenky et al., 2007; Yoshino et al., 2018; Katsyuba et al., 2020; Miller et al., 2020; Omran and Almaliki, 2020; Covarrubias et al., 2021). Although the NAD^+^-dependent sirtuins are suggested to play a key role in aging and NAD^+^ depletion-associated disorders, the precise roles of NAD^+^, sirtuins, and their downstream targets remain uncertain. Studying NAD^+^ homeostasis can be complicated by the dynamic flexibility of precursors cells use to generate NAD^+^. For example, NAM is both a NAD^+^ precursor and an inhibitor of NAD^+^-dependent enzymes such as sirtuins (Bitterman et al., 2002; Denu, 2003). As a result, NAM may modulate cellular function through pathways that depend on proper NAD^+^ homeostasis and/or sirtuin activity (Chong et al., 2002; Klaidman et al., 2003; Yaku et al., 2018; Katsyuba et al., 2020; Covarrubias et al., 2021). Therefore, it is important to understand the interconnections and cross-regulation of NAD^+^ precursors and associated cellular pathways. Studying factors that contribute to the regulation of NAD^+^ homeostasis may help understand the molecular basis of how NAD^+^ and NAD^+^ homeostasis factors regulate cellular function. This, in turn, may also help elucidate the roles of NAD^+^ metabolism in human disorders.

## 

### An Overview of NAD^+^ Biosynthesis Pathways and the Physiological Roles of NAD^+^

NAD^+^ biosynthesis pathways are largely conserved from bacteria to humans with a few species-specific differences. Overall, cellular NAD^+^ pool is maintained by three major pathways: 1) *de novo* synthesis; 2) salvage from NAM/NA; and 3) salvage from NR (Figure 1). These pathways converge at several different points and consume cellular pools of ATP, phosphoribosyl pyrophosphate (PRPP), and glutamine (Pinson et al., 2019; Croft et al., 2020). Specific NAD^+^ intermediates also contribute to other biosynthesis pathways or have signaling functions, and have diverse biological activities in humans (Kato and Lin, 2014; Tsang and Lin, 2015; Braidy and Grant, 2017; Cervenka et al., 2017; Yaku et al., 2018; Höglund et al., 2019; Pinson et al., 2019; Croft et al., 2020). Therefore, cells must maintain these metabolites in a controlled manner to promote their fitness and survival in response to various growth conditions.

#### *De novo* NAD^+^ Synthesis

The *de novo* pathway is also known as the kynurenine (KYN) pathway of tryptophan (TRP) degradation (Figure 1). In this pathway, the pyridine ring (nicotinamide, NAM) moiety of NAD^+^ is synthesized “*de novo*” from the amino acid TRP (as opposed to salvaging NAM from NAD^+^ intermediates). This is true for fungi, worms, flies, mammalian cells and some bacteria. However, for most bacteria and plants, aspartate (ASP) is used as the starting amino acid for *de novo* NAD^+^ synthesis. In yeast, this pathway is characterized by the synthesis of quinolinic acid (QA) from TRP by five enzymatic reactions mediated by Bna (biosynthesis of nicotinic acid) proteins (Bna2, Bna7, Bna4, Bna5, Bna1) and a spontaneous cyclization (Figures 2A,B) (Panozzo et al., 2002). Bna6 then transfers the phosphoribose moiety of PRPP to QA, which produces nicotinic acid mononucleotide (NaMN). The dual specificity NaMN/NMN adenylyltransferases (NMNATs), Nma1 and Nma2 in yeast, convert NaMN to nicotinic acid adenine dinucleotide (NaAD) by the addition of the AMP moiety of ATP (Emanuelli et al., 1999; Emanuelli et al., 2003). Finally, the glutamine-dependent NAD^+^ synthetase Qns1 converts NaAD to NAD^+^ (Bieganowski et al., 2003). Several steps in the *de novo* pathway require molecular oxygen as a substrate (Bna2, Bna4 and Bna1) (Figure 2A) (Panozzo et al., 2002). Therefore, cells grown under anaerobic conditions rely on the salvage pathways for NAD^+^ synthesis.

#### NA/NAM Salvage

NAD^+^ synthesis starting from NA is known as the Preiss-Handler pathway (Preiss and Handler 1958). In yeast, this pathway includes salvage of NAM, and therefore is also referred to as the NA/NAM salvage pathway (Figure 1 **)**. Under NA abundant conditions, such as in most yeast growth media, NA/NAM salvage is the preferred NAD^+^ biosynthesis route (Sporty et al., 2009). Consequently, genes of the *de novo* pathway (*BNA* genes) are silenced by the NAD^+^-dependent sirtuin Hst1 (Bedalov et al., 2003; James Theoga Raj et al., 2019). Conversely, NAD^+^ depletion results in decreased Hst1 activity leading to transcription activation of the *BNA* genes. NAM is deamidated by a nicotinamidase Pnc1 to produce NA (Ghislain et al., 2002) (Figure 1). Npt1 then converts NA to NaMN. NAM is produced from many NAD^+^ consuming reactions including sirtuin mediated protein deacetylation (Imai et al., 2000; Landry et al., 2000; Smith et al., 2000). Human cells do not have Pnc1-like nicotinamidases and therefore, NAM is mainly converted to NMN by NAM phosphoribosyltransferase (Nampt), an enzyme not found in yeast. NMN is then converted to NAD^+^ by NMNATs through the NR salvage branch (Figure 1). Interestingly, recent studies show that microbiota in the gut may assist the conversion of NAM to NA by bacterial nicotinamidases (Shats et al., 2020), which suggests NAM salvage to NA may also take place in humans with the aid of gut bacteria.

#### NR Salvage

NR is phosphorylated by the NR kinase, Nrk1, to produce NMN (Bieganowski and Brenner, 2004). NMNATs (Nma1, Nma2 and Pof1 in yeast) are responsible for the conversion of NMN to NAD^+^ (Emanuelli et al., 1999; Emanuelli et al., 2003; Kato and Lin, 2014). NR can also be converted to NAM. In yeast this is accomplished by nucleosidases Urh1 and Pnp1 and redirects NR to NA/NAM salvage (Belenky et al., 2007; Tempel et al., 2007) (Figure 1). Cells can also salvage NaR by converting it to NA or NaMN using the NR salvage pathway (Belenky et al., 2007). Recent studies have identified the reduced NR (NRH) as a new NAD^+^ precursor, which can be converted to reduced NMN (NMNH) by adenosine kinase to support NAD^+^ synthesis in mammalian cells (Giroud-Gerbetant et al., 2019; Yang et al., 2019; Yang et al., 2020). The NR salvage branch may confer flexibility in part due to compartmentalization of enzymes and precursors in this pathway (Lu and Lin 2011; Kato and Lin 2014; Croft, Venkatakrishnan et al., 2020). Moreover, yeast cells release and re-uptake small NAD^+^ precursors such as NA, NAM, QA and NR (Figure 1) (Kato and Lin 2014; Croft et al., 2018; James Theoga Raj et al., 2019). Specific transporters Tna1 (for NA and QA) (Llorente and Dujon, 2000; Ohashi et al., 2013) and Nrt1 (for NR) (Belenky et al., 2008) are responsible for the uptake of NAD^+^ precursors whereas the mechanisms of precursor release remain unclear. It is suggested that vesicular trafficking may also play a role in the movement of NAD^+^ metabolites (Kato and Lin, 2014; Croft et al., 2018; Croft et al., 2020).

#### Overview of Selected Disorders Associated with Altered NAD^+^ Metabolism

NAD^+^ and NAD^+^ metabolites play important roles in various cellular processes. Aberrant NAD^+^ metabolism, which causes substantial metabolic stress, has been implicated in diverse disorders ranging from obesity, diabetes, neuronal degeneration, kidney diseases, fatty liver disease and cancers. NAD^+^ preservation (or enhanced NAD^+^ homeostasis) is also shown to alleviate age-associated functional decline and/or extend life span in various model systems (Lin et al., 2000; Belenky et al., 2007; Balan et al., 2008; Lu et al., 2009; Cantó et al., 2012; Katsyuba et al., 2018; Mitchell et al., 2018; Yoshino et al., 2018; Fang et al., 2019; Okabe et al., 2019; Castro-Portuguez and Sutphin, 2020; Covarrubias et al., 2021). Recent discoveries and proposed molecular mechanisms of these diseases have been comprehensively discussed in several recent reviews (Yaku et al., 2018; Yoshino et al., 2018; Lautrup et al., 2019; Okabe et al., 2019; Katsyuba et al., 2020; Ralto et al., 2020; Covarrubias et al., 2021; Navas and Carnero, 2021). In this section, we will only briefly summarize selected studies of axonal degeneration, cancers and kidney disorders.

Axonal degeneration (Wallerian degeneration) is seen in many disorders in the nervous system including Alzheimers’s disease (AD), Parkinson’s disease (PD), and amyotrophic lateral sclerosis (ALS) (Brazill et al., 2017; Pehar et al., 2018; Jiang et al., 2020; Katsyuba et al., 2020). The discovery of the slow Wallerian degeneration mutant mouse (*Wld* ^*s*^) immediately linked NAD^+^ biosynthesis enzymes, NMNATs, with disease of the nervous system (Lunn et al., 1989; Mack et al., 2001). In *Wld* ^*s*^ mutant, NMNAT1 is fused with UBE4B, a ubiquitin conjugation factor. This change results in overexpression and redistribution of NMNAT1 to the cytoplasmic compartment of the axon. It is suggested that the *Wld* ^*s*^ NMNAT1 fusion protein protects axons through the synthesis of NAD^+^ (Conforti et al., 2009). However, it also appears that NMNATs may protect axons by functioning as protein chaperones (Zhai et al., 2008; Ocampo et al., 2013; Ali et al., 2016). Regarding the cause of neuronal death, one model suggests that the substrate of NMNATs, NMN, is neurotoxic because NMN may activate SARM1, triggering NAD^+^ destruction and production of ADP-ribose (ADPR), cyclic ADP-ribose (cADPR), and NAM, which all contribute to cell death (Essuman et al., 2017; Zhao et al., 2019). Another model suggests that NMNAT1 prevents SARM1-mediated NAD^+^ depletion (Gerdts et al., 2015; Sasaki et al., 2016). Although it is unclear whether it is NAD^+^ depletion or the accumulation of NMN that is the direct cause of neuronal death, it is well-supported that NAD^+^ depletion is the common factor shared in these two models.

NAD^+^ is an important driver of cancer cell metabolism and it has been observed that cancer cells have a higher NAD^+^/NADH ratio. This increased NAD^+^ fuels cancer cells by increased glycolysis leading to the “Warburg effect”: cancer cells prefer glycolysis and increased fermentation to lactate even in the presence of oxygen, leading to acidification of the tumor environment (Liberti and Locasale, 2016; Moreira et al., 2016; Yaku et al., 2018; Navas and Carnero, 2021). This acidification likely helps improve the cancer cell growth and immune suppression (Liberti and Locasale, 2016). NAD^+^ biosynthesis enzymes are candidates for targeting cancer metabolism. For example, over-expression of NAMPT, the rate-limiting factor for NAD^+^ synthesis in mammalian cells, is frequently observed in a number of malignant tumors including breast, colorectal, ovarian and prostate cancers (Yaku et al., 2018; Nacarelli et al., 2020; Navas and Carnero, 2021). NAMPT promotes cell proliferation by increasing the NAD^+^ pool, thus enhancing tumor progression and development (Garten et al., 2015; Sampath et al., 2015). The oncogene c-MYC was also reported to increase NAMPT expression in cancer cells (Cantor and Sabatini, 2012; Menssen et al., 2012; Yaku et al., 2018). NAMPT inhibition facilitates cancer cell killing likely because cancer cells have a higher demand for NAD^+^. In addition, since PARP mediated DNA repair requires NAD^+^, limiting NAD^+^ synthesis may also enhance cancer cell death (Kim et al., 2005). It has been shown that co-treatments of PARP inhibitors and NAMPT inhibitors induce synthetic lethality in breast cancer cells (Bajrami et al., 2012). In addition to PARP and NAMPT, other NAD^+^ homeostasis factors such as sirtuins and CD38 also play important roles in cancers and other metabolic diseases, which have been extensively discussed in recent reviews (Yaku et al., 2018; Yoshino et al., 2018; Heske, 2019; Konen et al., 2019; Okabe et al., 2019; Navas and Carnero, 2021).

Recent studies also show that the *de novo* NAD^+^ synthesis pathway plays an important role in the pathophysiology of kidney disorders including acute kidney injury (AKI) and chronic kidney disease (CKD) (Ralto et al., 2020). Mice with induced AKI displayed reduced quinolinate phosphoribosyltransferase (QPRT) activity (Figure 2B), while patients with renal ischemia also display accumulated QA (Poyan Mehr et al., 2018). It was hypothesized that the reduction of QPRT activity is likely associated with reduced NAD^+^ levels. Indeed, supplementation with NAM, metabolized independently of QPRT, was seen to improve renal function in cardiac surgery patients (Poyan Mehr et al., 2018). D*e novo* pathway activity is also reduced in chronic kidney disease (CKD). However, NR was ineffective in limiting experimentally induced CKD in mice, despite raising cellular NAD^+^ levels (Faivre et al., 2021). This may suggest that the *de novo* metabolites themselves have a role in the progression of CKD independent of NAD^+^ production.

At the molecular level, observed NAD^+^ depletion and dysregulation of cellular function are often associated with reduced NAD^+^ synthesis activity, increased NAD^+^ consuming activity (e.g. activated PARPs, CD38 and SARM1), diminished sirtuin signaling, mitochondrial dysfunction, oxidative stress and inflammation (Chini et al., 2020; Covarrubias et al., 2020; Jiang et al., 2020; Katsyuba et al., 2020; Covarrubias et al., 2021). Interestingly, specific NAD^+^ intermediates such as the KYN pathway metabolites (Figure 2B), have been linked to several human disorders seemingly independent of NAD^+^ levels (Schwarcz et al., 2012; Amaral et al., 2013; Cervenka et al., 2017). Recent studies have shown that inhibiting the activities of *de novo* pathway enzymes (Figure 2B), such as tryptophan-2,3-dioxygenase (TDO) (Breda et al., 2016) and kynurenine-3-monooxygenase (KMO) (Mole et al., 2016), may help alleviate specific neurological disorders. These strategies mostly center on increasing the ratio of neuroprotective kynurenic acid (KA) over neurotoxic QA or 3-hydroxykynurenine (3-HK) (Breda et al., 2016; Chang et al., 2018). Yeast cells also produce KA from KYN by the Aro8/9 kynurenine aminotransferase (KAT) (Figure 2B) (Ohashi et al., 2017). However, the function of KA in yeast remains unclear. The KYN pathway metabolites have also been shown to regulate various biological processes including immune cell response and host-pathogen signaling (Fallarino et al., 2012; Schwarcz et al., 2012; Amaral et al., 2013; Cervenka et al., 2017; Schwarcz and Stone, 2017), which are discussed in the next sections.

### Immunity, KYN Pathway Metabolites and *de Novo* NAD^+^ Metabolism

As briefly discussed above, KYN pathway metabolites play important roles in immune regulation. The synthesis of KYN pathway metabolites is also tightly controlled by the immune system (Fallarino et al., 2012; Schwarcz et al., 2012; Amaral et al., 2013; Cervenka et al., 2017; Schwarcz and Stone, 2017). Here, we further discuss the interconnections of the KYN pathway activity, inflammation, immunity, and NAD^+^ metabolism.

#### Activation of IDO1 and KYN Pathway During Inflammation

The first and rate-limiting step in the KYN pathway is the conversion of TRP to N-formylkynurenine (NFK), which is then converted to KYN and later other KYN metabolites (Figure 2B) (Wang et al., 2015; Yuasa and Ball, 2015). This NAD^+^ synthesis-associated TRP catabolism is initiated by tryptophan 2,3-dioxygenase (TDO) and/or indoleamine 2,3-dioxygenase (IDO). Among the many cell types that express *de novo* pathway enzymes, TDO is expressed highly in liver. There are two isoforms of IDO (IDO1 and IDO2) in mammals. *IDO1* is broadly expressed in various type of tissues whereas *IDO2* has a restricted pattern of expression (Cervenka et al., 2017; Schwarcz and Stone, 2017). The activity of IDO1, is stimulated by a variety of pro-inflammatory signals, chief among which is interferon-γ (IFN-γ). Mechanistically, IFN-γ downregulates the production of the adapter molecule DAP12, an event strongly associated with raised IDO1 activity in dendritic cells, leading to the endogenous production of KYN (Belladonna et al., 2008). IDO1 may also be activated to a comparatively lesser degree by a variety of pro-inflammatory cytokines and factors including lipopolysaccharide (LPS), tumor necrosis factor (TNF), platelet activating factor (PAF), and other interferons (Takikawa et al., 1986; Hassanain et al., 1993; Heyes et al., 1997; Pemberton et al., 1997; Munn, 2011; Guillemin, 2012; Smith et al., 2012; Song et al., 2017). It has been reported that TNF-α and IFN-γ cooperate synergistically in the promotion of IDO1 activity (Guillemin et al., 2001). On the other hand, IDO1 expression is reduced by anti-inflammatory cytokines such as interleukin 4 (IL-4) and interleukin 13 (IL-13) (Musso et al., 1994; Chaves et al., 2001). Kupffer cells were also seen to upregulate IDO1 in response to the administration of IFN-γ, leading to raised KYN levels (Yan et al., 2010). In addition, intracellular QA levels increase dramatically in response to immune stimulation in macrophages, microglia, dendritic cells, and other cells of the immune system. For example, injecting the hippocampus of rats with LPS results in a marked increase of QA levels in all compartments of the spleen that persists for several days. Injection of LPS also resulted in the recruitment of a large number of macrophages and microglia to the brain, where, surprisingly, very few cells displayed raised QA levels. This suggests that the brain balances the production of immunosuppressive KYN metabolites and the restoration of depleted NAD^+^ levels with the neurotoxic QA (Moffett et al., 2020). As precursors for NAD^+^, it is likely that a portion of QA and KYN metabolites are directed toward replenishing cellular NAD^+^ levels in response to inflammation and infection. Detailed mechanisms await to be further studied.

NAD^+^-consuming enzymes also play an important role in inflammation. The NAD^+^-dependent sirtuin proteins may serve as an example. One major feature of the inflammatory response is high energy consumption, which results in changes of the NAD^+^/NADH redox ratio. It has been shown that the sirtuins sense the changes of intracellular NAD^+^/NADH ratio to regulate inflammatory response (Liu et al., 2011; Smith et al., 2015; Chang et al., 2018). It has also been shown that NAMPT, the rate-limiting enzyme for NAD^+^ synthesis from NAM, is readily induced in neutrophils and macrophages by infections or pro-inflammatory cytokines and mediators (Jia et al., 2004). A switch of NAD^+^ synthesis from NAMPT-dependent salvage to IDO1-dependent *de novo* synthesis was observed in sustained immune tolerance. Mechanistically, it was suggested that activation of *de novo* NAD^+^ synthesis supplemented the nuclear NAD^+^ pool, which prolonged SIRT1 mediated repression of inflammatory gene (Zhang et al., 2019). CD38 is a major NAD^+^-consuming enzyme responsible for the age-related NAD^+^ decline (Camacho-Pereira et al., 2016; Tarragó et al., 2018). It has been shown that CD38 expression increases during aging in mouse metabolic tissues such as the white adipose tissue (WAT) and liver. Recent studies showed that inflammation increases CD38-mediated NAD^+^ degradation activity, which decreases NAD^+^. The increase in CD38 in metabolic tissues during aging is likely mediated by accumulation of pro-inflammatory M1-like macrophages that also express CD38 (Chini et al., 2020; Covarrubias et al., 2020).

In immune cells, as in most non-liver tissues, TRP catabolism is initiated by IDO. This enzyme is ubiquitously expressed and has affinity for substrates other than TRP, including 5-hydroxytryptophan and serotonin (Cervenka et al., 2017). IDO1-mediated TRP catabolism in the host microenvironment occupied by parasites, viruses, and bacteria has been seen as a way to curb their proliferation (Pfefferkorn, 1984). However, immune cells can also contribute to TRP degradation during nonpathogenic inflammation, indicating that IDO1 has a broader spectrum of activity on immune cell regulation (Schröcksnadel et al., 2006). Since an unrestrained immune response is detrimental to cells, IDO1 expression is highly regulated in the immune system. IDO1 expression is stimulated by pathogens- and host-derived signals including pro-inflammatory cytokines (e.g. IFN-γ) and endotoxins (e.g. LPS), which can also be inhibited by anti-inflammatory cytokines (Divanovic et al., 2012; Suzuki et al., 2012; Wang et al., 2012). IDO1-dependent TRP degradation promotes an immunosuppressive environment by the production of TRP metabolites with immune activity, as well as by triggering an amino acid-sensing signal in cells due to TRP depletion (Cervenka et al., 2017). Therefore, this pathway has emerged as a rate-limiting step for metabolic immune regulation.

#### Immune Activation and Suppression by the KYN Pathway Metabolites

Control of TRP metabolism by IDO in dendritic cells (DCs) has been suggested to be a regulator of innate and adaptive immune responses (Fallarino et al., 2012). KYN metabolites suppress the activity of various immune cells including dendritic cells and macrophages in mice (Fallarino et al., 2012). Antigen tolerance is mediated by IDO1 activity in T cells (Munn et al., 1998) as well as in dendritic cells (Grohmann et al., 2000). It has been shown that both KYN metabolites and IDO1 can initiate tolerogenesis by dendritic cells and that IDO-mediated KYN production in DC leads to the proliferation of regulatory T cells (Tregs) (Belladonna et al., 2006; Belladonna et al., 2008; Fallarino et al., 2012). Positive feedback also occurs wherein IDO1 activity in DCs promotes the emergence of regulatory T cell phenotypes in CD4^+^ T cells, which itself then stimulates further IDO1 activity in DCs (Hill et al., 2007; O'Farrell and Harkin, 2017). It is suggested that these effects are mediated at least partly by KYN activation of aryl hydrocarbon receptor (AhR), a ligand-activated transcription factor expressed in cells of both innate and adaptive immune systems (Mezrich et al., 2010). Several KYN pathway metabolites, including KYN, 3-HK, and 3-hydroxyanthranilic acid (3-HA), promote apoptosis and consequently may serve to combat the proliferation of infectious pathogens (Kwidzinski and Bechmann, 2007). 3-HA also promotes the production of transforming growth factor b (TGF-β), which further drives T cells toward regulatory phenotypes (Munn, 2011; O'Farrell and Harkin, 2017). 3-HA also appears to suppress glial cytokine expression during inflammation, while it increases the expression of the antioxidant and anti-inflammatory enzyme, hemeoxygenase-1 (Krause et al., 2011).

The stimulation of KYN pathway activity during inflammation also depletes cellular TRP stores and thereby reduces the amount available for conversion to other metabolites such as serotonin and melatonin (Höglund et al., 2019). Infections, stress, and dietary intake all contribute to the usage of TRP in KYN metabolism, limiting its availability for serotonin biosynthesis (O'Farrell and Harkin, 2017). As a neurotransmitter, serotonin is involved in the regulation of the central nervous system, the cardiovascular system, and many other processes in the body (Berger et al., 2009), while melatonin affects a range of phenotypes including oxidative stress response, mitochondrial metabolism (Reiter et al., 2018), and circadian rhythms (Emens and Burgess, 2015). Moreover, a recent study in yeast showed that overexpression of *BNA2* (*BNA2*-oe) increased flux through the TRP-producing shikimate/aromatic amino acid biosynthesis pathways, leading to reduced lipid droplet formation in aging cells due to depletion of necessary precursors (Beas et al., 2020). Interestingly this study also showed that *BNA2-*oe-induced life span extension and reduced lipid droplet formation is independent of NAD^+^ production.

#### Physiological Roles of the KYN Pathway Metabolites

Aberrations in KYN pathway metabolites are found in a variety of diseases and are often related to inflammation and oxidative stress in the affected tissues. Dysregulation of these metabolites has been implicated in neurodegenerative and neurological disorders, as well as in psychiatric diseases such as depression and schizophrenia (Fallarino et al., 2012; Schwarcz et al., 2012; Amaral et al., 2013; Cervenka et al., 2017; Schwarcz and Stone, 2017). For example, QA, KA, and 3-HK, have all been shown to be related in some capacity to neurological health (Breda et al., 2016; Chang et al., 2018). QA is neurotoxic, an agonist of the NMDA receptor, which ordinarily binds glutamate. High levels of QA result in hyperactivation of this receptor and excitotoxicity, as well as glutamate toxicity due to excessive glutamate release from astrocytes and inhibited glutamine synthetase function (Guillemin, 2012). QA in complex with Fe^3+^ also results in oxidative damage to lipids (Goda et al., 1996; Stípek et al., 1997; Guillemin, 2012). Elevated QA levels have previously been identified in cases of HIV-associated neurological damage (Heyes et al., 2001), Alzheimer's disease (Guillemin et al., 2003), multiple sclerosis (Aeinehband et al., 2016). Conversely, KA is generally neuroprotective, tending to decline in Huntington's disease (Beal et al., 1992). The effect of KA opposes that of QA in acting as an antagonist of the NMDA receptor and other glutamate receptors, as well as of the α-7 nicotinic acetylcholine receptor (Wirthgen et al., 2017). However, raised KA levels are also associated with neurological dysfunction, seen in a variety of cases ranging from Alzheimer's disease (Baran et al., 1999) to Down's syndrome (Baran et al., 1996). Increased 3-HK levels are associated with Alzheimer's disease (Lewitt et al., 2013) and, like QA, 3-HK's neurotoxic effects are associated with free radical generation (Okuda et al., 1996). Indeed, elevated levels of both 3-HK and QA are a feature of Huntington's disease as well (Guidetti et al., 2006; Thevandavakkam et al., 2010; Mason and Giorgini, 2011). 3-HK and 3-HA, however, have also been shown to reduce the cytokine-induced destruction of neurons (Krause et al., 2011). It is therefore important for cells to effectively regulate flux through the KYN pathway and balance the levels of each intermediate produced.

Alterations in the KYN pathway metabolism have far-ranging effects on many other aspects of host health as well. The KYN pathway has, for instance, a significant influence on the liver. Activation of lipid oxidation and mitochondrial proliferation in the livers of rats resulted in increased serum levels of TRP, downstream KYN metabolites, and NAM, altogether indicating that mitochondrial activity in hepatocytes is strongly correlated with *de novo* biosynthesis of NAD^+^. It was also seen however that this resulted in reduced levels of IDO1 expression, while the KYN/TRP ratio was negatively correlated with mitochondrial function (Lindquist et al., 2020); this suggests other forms of TRP metabolism may also play a role. Further, heightened hepatic and adipose tissue expression of IDO1 is observed in obese individuals (Wolowczuk et al., 2012). Moreover, liver has a central role in modulating systemic TRP levels because hepatocytes are the only cell types that contain all the components for any branch of KNY metabolism (Moffett and Namboodiri, 2003).

IDO1 expression is limited to particular cells types, among which are a variety of immune cells described in the beginning of this section, as well as the smooth muscle cells of the cardiovascular system (Song et al., 2017). A second IDO isoform, IDO2, is expressed in the human liver, spleen, kidney, and brain, though not in the heart (Metz et al., 2007; Song et al., 2017). IDO2 appears to be constitutively expressed and does not respond to inflammatory signals in the manner of IDO1 (Prendergast et al., 2014). The reliance of the heart and vasculature on IDO1 may make the influence of the immune system on cardiovascular *de novo* metabolism activity particularly significant. IDO1 is also expressed in tumors and lymph nodes, which help create an immunosuppressive microenvironment both by depleting TRP and by accumulating immunosuppressive KYN metabolites. It is suggested that IDO1 expression produced KYN metabolites contribute to the escape of the immune response by binding to and activating AhR, a primary receptor of KYN metabolites (Opitz et al., 2011; Navas and Carnero, 2021). High levels of IDO, has also been detected in many types of tumors associated with poor response. Owing to its role in immunosuppression, IDO has also been proposed to be targets of cancer therapy (Löb et al., 2009; Brochez et al., 2017).

### Impact of Infection on KYN Pathway Metabolites and NAD^+^

#### Infection and the KYN Pathway Metabolites

It has been increasingly seen that KYN pathway metabolite homeostasis is disrupted during infection. As noted prior, there is a well-established association between host immunity and flux through the KYN pathway, while several pathogens themselves and their mechanisms of infection have also been shown to induce alterations in KYN pathway metabolism. Most studies centered on the toxicity, protective effects, and/or immunosuppressive effects of the *de novo* KYN pathway metabolites Figure 2B. It is unclear whether NAD^+^ levels were also significantly altered in the host cells by specific pathogens in some of these studies.

*Toxoplasma gondii* appears to reduce traffic through the *de novo*KYN pathway by the phosphorylation-mediated degradation of IDO1. The mechanism appears to involve AKT-mediated phosphorylation signaling cascade. After infection by *T. gondii,* IDO1 levels decline, presumably resulting in reduced levels of KYN. It appears that supplementation with KYN and the KYN analog teriflunomide hinders the establishment of *T. gondii* infection, as does the production of free radicals (Majumdar et al., 2019). As noted prior, the KYN pathway intermediates 3-HK and QA are known to stimulate free radical production. This leaves the question of whether and in what manner KYN pathway activity may be protective against toxoplasmosis, which, like perturbations of KYN pathway metabolism, is often associated with neurological disorders (Abo-Al-Ela, 2020). 3-HK has been shown to stimulate apoptosis by the production of oxidative stress, the crosslinking of proteins, and the inhibition of the respiratory electron transport chain (Crozier-Reabe et al., 2008). Majumdar et al. hypothesize that the activation of apoptotic pathways by KYN metabolites is one factor responsible for host resistance to *T. gondii* infection (Majumdar et al., 2019).

Infection by Borna disease virus (BDV) also results in dysfunctional KYN pathway metabolism. BDV, like *T. gondii*, is well known to cause neurological damage. Formisano, et al. investigated the BDV-alone vs. immune-mediated consequences of infection by examining both adult and neonatal rats (Formisano et al., 2017). The authors identified a modest increase in IDO1 expression in the cerebellum and hippocampus of neonatal rats during infection, while immune competent adult rats show a marked increase of IDO1 expression in the cerebellum and hemispheres of the brain. Expression of KATII, the main enzyme of neural KYN biosynthesis, is increased in the brain tissue of neonatal, but not adult rats. Levels of KYN monooxygenase (KMO), which produces 3-HK from KYN (Figure 2B), in both adult and neonatal rats are elevated compared to mock-infected animals. Neonatal rats exhibit raised levels of QA in the hippocampus and striatum, with no changes of KYN levels in brain tissue (Formisano et al., 2017). This may hint at a possible consequence of BDV-altered KYN metabolism being the production of excess neurotoxic QA. In any event, an immune-independent role of BDV in the manipulation of KYN pathway metabolism is clear. In addition, both adult and neonatal rats experience adverse neurological effects from BDV infection. It is therefore likely that at least part of the means by which BDV harms neurological health may occur by way of direct influence on KYN pathway metabolism, with different effects observed depending on immune mobilization.

Aberrations of the KYN pathway activity also appear to be associated with infection by the SARS-CoV-2 coronavirus responsible for COVID-19. Raised serum levels of *de novo* KYN and KA were noted in COVID-19 patients which, interestingly, correlated with serum levels of interleukin-6 (IL-6), a hallmark of SARS-CoV-2 infection. This relationship may be explained in part by the pro-inflammatory character of several KYN metabolites, or by the stimulation of certain KYN pathway enzymes (e.g. IDO1 activity promoted by IFN-γ) by inflammation. This is concomitant with reduced levels of TRP and its other metabolites, such as serotonin, suggesting that cellular TRP stores are shunted through the KYN pathway in SARS-CoV-2 infected cells, which may be related to the "cytokine storm" seen in severe cases (Thomas et al., 2020).

The KYN pathway also appears to play a key role in human immunodeficiency virus (HIV) pathogenesis. An HIV-infected group of Subsaharan Africans showed altered KYN pathway metabolism relative to a non-infected control group, displaying reduced TRP levels, raised KYN and NAM levels and, most notably, an approximately 20-fold increase of QA (Bipath et al., 2015). Increases of cellular QA concentration, though less significant, have also been associated with HIV infection elsewhere, with the distinction that the groups surveyed were drawn from developed countries (Hayes, 1989; Look et al., 2000; Heyes et al., 2001). QA, being a potent neurotoxin, may in part explain the neurological damage associated with the progression of HIV infection (McArthur et al., 2010). Raised KYN concentration is also negatively correlated with CD4 levels (Bipath et al., 2015; Kardashian et al., 2019). The KYN/TRP ratio in HIV-infected women is increased relative to healthy volunteers, rises with age, and is negatively correlated with platelet count (Kardashian et al., 2019). In contrast to its protective effect against neurological problems, a high KYN/TRP ratio appears to be associated with aging and the progression of HIV, though of course this is confounded by a likely concomitant rise of QA. Altogether, this suggests a mechanism by which HIV promotes flux through the KYN pathway, thereby increasing the cellular levels of these KYN metabolites.

Hepatitis C virus (HCV) is also known to dysregulate KYN pathway metabolism during infection. In patients coinfected with HIV and HCV, levels of KYN, an immunosuppressant, are significantly elevated, together with the KYN/TRP ratio. Raised KYN levels are also positively correlated with fibrosis of the liver and with insulin levels under these conditions. After successful HCV treatment with IFN-α/ribavirin, KYN levels remained raised (Jenabian et al., 2016). Another group confirmed that HIV/HCV coinfection raises the KYN/TRP ratio relative to other surveyed groups (non-infected, HIV monoinfected) and that this ratio is positively correlated with liver stiffness (Kardashian et al., 2019). HCV patients with and without cirrhosis also display increased levels of IDO1 activity, which appear to stabilize after treatment (Larrea et al., 2007).

Further, a link has been established between the development of several severe viral pathologies of the central nervous system and dysregulated KYN metabolism. The development of subacute sclerosing panencephalitis (SSPE) after measles infection was associated with significantly raised QA levels in cerebrospinal fluid. Patients with bacterial and viral meningitis displayed even more drastic phenotypes, with QA levels raised by approximately an order of magnitude compared to uninfected control patients. SSPE patients, however, did not show significantly altered KYN/TRP ratios (Inoue et al., 2020). Moreover, infection of mice with hamster neurotropic measles virus leads to the development of encephalitis as well as a marked increase in levels of QA and 3-HK (but again, not KYN) in the hippocampus (Lehrmann et al., 2008).

A significant number of infectious fungi have also been noted to alter host IDO activity, both positively and negatively, suggesting a nexus between these infectious agents, KYN/TRP metabolism, and the host immune response (Choera et al., 2017). Interestingly, it has recently been discovered that loss of the native IDO genes of the fungus, *Aspergillus fumigatus,* redirects TRP catabolism into a pathway, mediated by the aromatic aminotransferase AroH, which generates indole acetate and indolepyruvate (Choera et al., 2017). Mice were also shown to be more vulnerable to infection by *A. fumigatus* fungi lacking IDO, while mice without IDO1 displayed a similarly increased susceptibility to infection. The authors also noted raised inflammation after infection with fungi lacking IDO genes vs. wild-type controls, which was hypothesized to be due to production of AhR ligands by way of the indole pathway. Indeed, deletion of AroH/I reduces the virulence of *A. fumigatus.* Altogether, this suggests a state of homeostasis evinced between the host and fungus, downregulating immune signaling and inflammation through KYN pathway activity (Zelante et al., 2021).

#### Infection and NAD^+^

Beyond the production of KYN metabolites, NAD^+^ metabolism as a whole is also notably responsive to various infections. Recently, altered NAD^+^ levels have been associated with SARS-CoV-2 infection and the disease state observed in COVID-19. Using mouse hepatitis virus, a model coronavirus, Heer et al. show that infection reduces NAD^+^ levels. They also note that expression of numerous PARPs, along with more modest effects observed for several sirtuins and IDO1/IDO2, is increased in several cell lines infected with SARS-CoV-2 (Heer et al., 2020). It is speculated that SARS-CoV-2 depletes cellular NAD^+^ levels by overexpressing a set of PARPs, all of which compete for the same NAD^+^ pool. The authors determined that PARP10 activity is not increased by inhibition of the main human PARPs, PARP1/2, but is increased by overexpression of NAMPT (and thereby raising NAD^+^ levels) (Heer et al., 2020). This effect may be due to competition with other NAD^+^ consuming enzymes, including the remaining PARPs and a variety of sirtuins. Altogether, it appears that SARS-CoV-2 infection induces high expression of a set of NAD^+^ consuming enzymes, which ultimately reduces NAD^+^ levels. *In silico* modeling of the main SARS-CoV-2 protease indicates that NAD^+^ derivatives may bind to its active site and inhibit its activity, thereby hindering modification of the SARS-CoV-2 replicase and the replication of the virus RNA genome (Martorana et al., 2020). This may be a factor responsible for the particular danger the virus poses to the elderly (Perrotta et al., 2020), as NAD^+^ levels decline consistently with age. Indeed, Omran and Almaliki (Omran and Almaliki, 2020) have speculated about a relationship between the reduction of NAD^+^ levels with age and the increased susceptibility to COVID-19 that is exhibited by this population. Loss of cellular NAD^+^ corresponds to reduced efficacy of PARP-dependent DNA repair and sirtuin-dependent telomere stabilization, both of which are associated with aging. In addition, NAD^+^ metabolites have an extensive interface with immune signaling (Omran and Almaliki, 2020). Miller et al. (2020) also note that reduced NAD^+^ levels may at least in part explain the heightened mortality rate of COVID-19 not only in elderly patients, but also those with comorbidities such as diabetes, hypertension, and obesity. They note several factors that may be responsible: increased CD38 expression, associated with insulin resistance, leading to lowered NAD^+^ levels; increased oxidative stress leading to increased PARP activity, and consequently reduced NAD^+^ pools; and also a reduction of sirtuin activity resulting in limitation of autophagy and derepression of inflammatory signals (Miller et al., 2020). It has also been suggested that SARS-CoV-2 infection may lead to activation of PARP1, thereby depleting the NAD^+^ pool, and that a possible avenue of treatment for this is the PARP (and sirtuin) inhibitor, NAM (Badawy, 2020). Conversely, increased levels of NA have also been associated with infection by SARS-CoV-2 (Thomas et al., 2020).

*Mycobacterium tuberculosis* also targets cellular NAD^+^ pools during infection. The tuberculosis necrotizing toxin (TNT), responsible for the death of infected macrophages, acts by hydrolyzing NAD^+^ and depleting stores in the cell. TNT is the C-terminal domain of the outer membrane protein CpnT (Sun et al., 2015). As macrophage death cannot be induced by TNT defective for this NAD^+^ glycohydrolase activity, it is likely that this is a primary mechanism by which necroptosis occurs in tuberculosis (Sun et al., 2015). It was further shown that NAD^+^ depletion by TNT is sufficient to cause macrophage death, leading to depolarization of the mitochondrial membrane and necroptosis. Replenishing NAD^+^ by NAM supplementation was sufficient to reduce necroptosis and increase macrophage viability after infection by *M. tuberculosis* (Pajuelo et al., 2018). TNT is also involved in the production of reactive oxygen species (ROS), a hallmark of *M. tuberculosis* infection. This was shown to be dependent on its NAD^+^-glycohydrolase activity and the depletion of cellular NAD^+^ pools, with NAM supplementation proving effective in reducing ROS accumulation after infection with strains expressing functional CpnT/TNT (Pajuelo et al., 2020). It therefore appears that the depletion of NAD^+^ is a major strategy employed during *M. tuberculosis* infection, which may be alleviated by targeting the replenishment of NAD^+^.

### Regulation of *de Novo* NAD^+^ Metabolism

The foregoing discussion has attempted to identify the reciprocal connections between various signaling pathways and the production of KYN pathway metabolites, with a particular focus on their modulation by infection, whether due to inflammatory stress produced by infection or hijacking of host signaling by pathogens. It now remains to trace a general network of the signals known to affect KYN pathway activity and associated *de novo* NAD^+^ metabolism. Several NAD^+^ homeostasis regulatory factors have been identified in yeast, which include transcriptional control, feedback inhibition, nutrient sensing, and enzyme or metabolite compartmentalization (Anderson et al., 2003; Bedalov et al., 2003; Gallo et al., 2004; Bieganowski et al., 2006; Medvedik et al., 2007; Lu et al., 2009; Lu and Lin, 2011; Kato and Lin, 2014; Croft et al., 2018; James Theoga Raj et al., 2019; Pinson et al., 2019). In this section, we discuss stress and nutrient signaling pathways that have been suggested to modulate *de novo* NAD^+^ metabolism in yeast and in higher eukaryotes. Some of these factors may serve as potential targets of infectious pathogens and immune stimulation.

In budding yeast, *de novo* NAD^*+*^ biosynthesis activity is normally repressed under NAD^+^ repleted conditions wherein NA/NAM salvage activity is high. Silencing of the *de novo* pathway *BNA* genes is mediated by the NAD^+^-dependent sirtuin Hst1 and associated co-repressor complexes. During NAD^+^ depletion, the *BNA* genes are de-repressed due to loss of Hst1 activity leading to NAD^+^ synthesis from the *de novo* branch (Bedalov et al., 2003; James Theoga Raj et al., 2019). *De novo* NAD^+^ biosynthesis is also shown to be stimulated by adenine limitation (Pinson et al., 2019). This occurs due to flux through the *de novo* adenine biosynthetic pathways resulting in raised levels of the intermediate 5-aminoimidazole-4-carboxamide ribonucleotide (AICAR). AICAR then promotes Bas1-Pho2 complex formation, which serves as a transcriptional activator for the BNA genes of *de novo* NAD^+^ biosynthesis (Pinson et al., 2019). This links *de novo* NAD^+^ production not only to purine metabolism, but possibly to phosphate sensing as well, as AICAR also promotes the formation of the Pho2-Pho4 complex (Pinson et al., 2009), a transcriptional activator of *PHO* pathway targets, which are expressed under phosphate depleted conditions. The sharing of Pho2 between these two complexes is a particularly interesting point which may have implications for the sensitivity of *de novo* NAD^+^ metabolism to cellular phosphate levels.

Activity of *de novo* NAD^+^ metabolism is also known to be sensitive to levels of micronutrients such as certain metal ions. Both copper depletion and copper stress in particular were seen to elevate *BNA* gene expression above levels observed under standard conditions (Cankorur-Cetinkaya et al., 2016; James Theoga Raj et al., 2019). In the latter case this is likely mediated by the copper-sensing transcription factor Mac1, here serving as a co-repressor, which is inhibited in the presence of excess copper (Keller et al., 2005). The causal influences underlying increased *BNA* expression under copper depleted conditions are more elusive and likely do not depend on Mac1 given its propensity for activity under copper limiting conditions. However, iron transport in *S. cerevisiae* depends on cellular copper levels, which results in reduced intracellular iron levels in the absence of copper (Yuan et al., 1995; De Freitas et al., 2003). This may therefore indicate a potential link between iron homeostasis and *de novo* NAD^+^ metabolism as well. In fact, it has been observed that deletion of *CCC2*, an intracellular copper transporting ATPase, leads to raised *BNA2* and *BNA4* expression under low copper conditions and reduced levels under high copper conditions. Deletion of another copper chaperone protein, *ATX1*, produces opposite results, wherein *BNA* induction occurs in copper-rich conditions but is repressed under copper-limited conditions (Cankorur-Cetinkaya et al., 2016). Both Atx1 and Ccc2 are required for the mode of copper transport that later facilitates iron transport. Atx1 is a copper chaperone that passes copper from Ctr1, its membrane transporter, to Ccc2, where it is later inserted into Fet3 in order to enable high affinity iron transport (Lin et al., 1997). Wild type cells conversely show induced *BNA* expression under both conditions (James Theoga Raj et al., 2019). This is especially interesting in light of the requirement of iron for the catalytic activity of 3-hydroxyanthranilate-3,4-dioxygenase (HAAO; human homolog of Bna1) (Zhang, Colabroy et al., 2005) and the marked stimulation of Bna1 activity by iron supplementation (Stachowski and Schwarcz, 2012), together with the generation of oxidative stress by QA in the presence of iron (Pláteník et al., 2001), the latter of which in particular may make the down-regulation of *de novo* activity in the presence of iron (corresponding to up-regulation in its absence) a significant factor for cell function. Both copper and iron are capable of producing oxidative stress (De Freitas et al., 2003), making the homeostasis of these two metals, considered together with oxidative *de novo* pathway intermediates like the 3-HK or the aforementioned QA, an important factor to regulate. The connection between *de novo* and metal ion homeostasis is therefore significant and may be an additional factor contributing to the dysregulation of *de novo* metabolism in disease states.

The *de novo* pathway is inactive under anoxic conditions, due to the oxygen dependence of the reactions mediated by Bna2, Bna4, and Bna1 (Panozzo et al., 2002; Perli et al., 2020). It is also known that heme is necessary for the activity of mammalian IDO. Heme appears to be a limiting factor for *de novo* NAD^+^ biosynthesis, as heme titration by apo-myoglobin significantly reduces IDO1 activity (Nelp et al., 2018), which mediates the rate limiting step of *de novo* metabolism (Dey et al., 2017). IDO1 binding to heme is also influenced by iron, with iron supplementation significantly raising IDO1 activity (Donley et al., 2019). Moreover, the oxidation state of iron is also an important factor in IDO1-heme interaction, with IDO1 showing approximately 10-fold greater affinity for heme in the presence of ferrous (Fe^2+^) vs. ferric (Fe^3+^) iron (Nelp et al., 2018). This may possibly lead to downregulation of *de novo* metabolism under conditions of oxidative stress, a strategy that would prevent the further production of oxidative *de novo* intermediates such as 3-HK and QA. IDO2 may also negatively regulate *de novo* metabolism activity in some contexts due to competition with IDO1 for heme (Lee et al., 2014). Like IDO1 (Nelp et al., 2018), Bna2 may also require heme for its function due to the high degree of homology between the two enzymes and the ability of IDO1 to complement Bna2 function in budding yeast (Cerejo et al., 2012). This would indicate a close link between *de novo* metabolism and mitochondrial respiration, both of which require oxygen and, putatively, heme. Indeed, Bna4 localizes to the mitochondrial outer membrane and links *de novo* NAD^+^ metabolism with mitochondrial function (Braun, 2012). Deletion of KMO (yeast Bna4) was seen to suppress polyQ-mediated cytotoxicity (Giorgini et al., 2005). It appears that this is dependent upon the accumulation of *de novo* intermediates downstream of Bna4, namely 3-HK and QA (Giorgini et al., 2005). Bna4 inhibition reduces the levels of these compounds as well as cytotoxicity and production of ROS (Giorgini et al., 2008). Moreover, in mouse models of Alzheimer's and Huntington's diseases, the inhibition of KMO (Bna4 in yeast) was confirmed to protect against neurodegeneration (Mason and Giorgini, 2011; Zwilling et al., 2011).

A genetic screen also revealed that Bna4 is a flavoprotein, binding to flavin adenine dinucleotide (FAD^+^) (Gudipati et al., 2014). This may link the biosynthesis of FAD^+^ and NAD^+^ and make *de novo* NAD^+^ biosynthesis sensitive to cellular FAD^+^ levels, consequently integrating the factors involved in the regulation of FAD^+^ metabolism with the regulation of NAD^+^ metabolism. Interestingly, NAD^+^ and FAD^+^ metabolism have elsewhere been seen to be connected; it is known that NAD^+^ inhibits the activity of FAD pyrophosphatase and prevents FAD degradation (Giancaspero et al., 2013). Pyridoxal 5-phosphate (PLP) is also known to be required as a cofactor for kynureninase (Bna5) (Panozzo et al., 2002; Perli et al., 2020) and KAT (Bna3) (Wogulis et al., 2008; Perli et al., 2020). Although the latter is not strictly part of the *de novo* pathway, it can convert KYN to KA (Figure 2B) and may therefore influence the levels of *de novo* intermediates and flux through the pathway. This could potentially connect *de novo* metabolism with factors involved in pyridoxal biosynthesis, which requires intermediates of glycolysis and the pentose phosphate pathway, along with ammonia (di Salvo et al., 2011). Pyridoxal kinase, involved in pyridoxal salvage by phosphorylating it to PLP (along with the respective phosphorylation of pyridoxine and pyridoxamine), is inhibited by a variety of common compounds, among which are caffeine, theobromine (Ubbink et al., 1990), dopamine (Lainé-Cessac et al., 1997), and various pharmaceuticals (di Salvo et al., 2011). The requirement of PLP as a cofactor of kynureninase (Kynu) (Lima et al., 2007) and KYN aminotransferase (KAT) (Rossi et al., 2004) is conserved in human. The pathways enumerated above may therefore indicate some avenues by which a ramifying network of signals is connected with the regulation of *de novo* NAD^+^ metabolism.

## Conclusion and Perspective

NAD^+^ biosynthesis is governed by a complex and interconnected network of metabolic and cellular signals. These pathways may be hijacked and modified by a variety of infections agents, therefore having widely ramifying effects on cellular health. Targeting particular features of NAD^+^ metabolism may then be an effective therapeutic strategy to ameliorate the virulence of certain pathogens. The *de novo* NAD^+^ biosynthesis/KYN pathway has a particularly close relationship with mounting responses against infection due to the immune signaling properties of its intermediates. Not only may KYN metabolites up- or down-regulate immune activity under various conditions, but KYN pathway activity in turn may be influenced by immune signaling, as in the cytokine-dependent activation of IDO1, responsible for catalyzing the rate-limiting and initial step of the pathway.

Flux through the KYN pathway, and through NAD^+^ metabolism *in toto*, may be influenced by a supra-organismal pool of metabolites drawn from the host, the pathogen, and even the members of the gut microbiota. Moreover, each site of NAD^+^ production during infection will be governed by a large and circumstantially unique signaling network, modulated by complex metabolic and environmental factors. Consequently, it is of immense practical and scientific interest to understand the dynamic and varied relationships that link infection status with NAD^+^ metabolic activity. Overall, recent studies have shown that NAD^+^ metabolism is an emerging therapeutic target for metabolic disorders as well as infections. Understanding the regulation and interconnections of NAD^+^ metabolites may help elucidate the complex mechanisms regulating NAD^+^ homeostasis. These studies may also contribute to the development of effective NAD^+^-based therapeutic strategies specific for different types of NAD^+^ deficiency related disorders.
